# Association of genetically predicted blood sucrose with coronary heart disease and its risk factors in Mendelian randomization

**DOI:** 10.1038/s41598-020-78685-5

**Published:** 2020-12-09

**Authors:** Ting Zhang, Shiu Lun Au Yeung, C. Mary Schooling

**Affiliations:** 1 School of Public Health, Li Ka Shing Faculty of Medicine, The University of Hong Kong, Hong Kong SAR, China; 2 CUNY School of Public Health and Health Policy, New York, USA

**Keywords:** Behavioural genetics, Cardiovascular diseases, Risk factors

## Abstract

We assessed the associations of genetically instrumented blood sucrose with risk of coronary heart disease (CHD) and its risk factors (i.e., type 2 diabetes, adiposity, blood pressure, lipids, and glycaemic traits), using two-sample Mendelian randomization. We used blood fructose as a validation exposure. Dental caries was a positive control outcome. We selected genetic variants strongly (*P* < 5 × 10^–6^) associated with blood sucrose or fructose as instrumental variables and applied them to summary statistics from the largest available genome-wide association studies of the outcomes. Inverse-variance weighting was used as main analysis. Sensitivity analyses included weighted median, MR-Egger and MR-PRESSO. Genetically higher blood sucrose was positively associated with the control outcome, dental caries (odds ratio [OR] 1.04 per log_10_ transformed effect size [median-normalized standard deviation] increase, 95% confidence interval [CI] 1.002–1.08, *P* = 0.04), but this association did not withstand allowing for multiple testing. The estimate for blood fructose was in the same direction. Genetically instrumented blood sucrose was not clearly associated with CHD (OR 1.01, 95% CI 0.997–1.02, *P* = 0.14), nor with its risk factors. Findings were similar for blood fructose. Our study found some evidence of the expected detrimental effect of sucrose on dental caries but no effect on CHD. Given a small effect on CHD cannot be excluded, further investigation with stronger genetic predictors is required.

## Introduction

Sugar, a common component of diet, is known to cause dental caries^1^, and has been thought to have adverse health effects, particularly on obesity and type 2 diabetes (T2D) for decades^2^. Whether sugar causes cardiovascular disease (CVD) was heavily debated in the context of whether dietary sugar or fat contributed to the rise in coronary heart disease (CHD) in the mid to late twentieth century in many Western countries^3,4^. Despite some initial concern about the role of protein^5^, dietary guidelines since the 1980s have largely focused on promoting a low-fat, high-carbohydrate diet to lower blood cholesterol and prevent CVD^5^. However, with the upward trend in obesity and T2D^6^ the role of sugar is being reappraised. In 2015, the World Health Organization (WHO) recommended limiting free sugar intake to less than 10% of total energy intake^7^.

Observationally, systematic reviews and meta-analyses of prospective cohort studies suggest associations of sugar intake, mostly in the form of sugar sweetened beverages (SSB), with higher risks of CHD^8^, T2D^9^, weight gain^10,11^, hypertension^12^, and elevated triglycerides, low-density lipoprotein cholesterol (LDL-C) and blood glucose^13^. However, observational studies, largely from Western settings, may be biased by confounding by socioeconomic position and lifestyle^14^. In contrast, our previous findings from a birth cohort of Hong Kong Chinese children, where adiposity is not strongly confounded by socio-economic position^15^, suggested no association of SSB consumption with adiposity^16^. Randomized controlled trials (RCT) of the effect of sugar intake on CVD or T2D are lacking. Meta-analyses of RCTs on adiposity tend to show a positive effect of sugar or SSB consumption on weight gain in trials of ad libitum diets^10,11^, but not in trials of isoenergetic exchange of sugar with other carbohydrates^10,17^. Most of the trials were small and short-term^10,11,17^, with a meta-analysis possibly biased by publication bias and heterogeneity^10^. Evidence from RCTs on the effects of sugar intake on blood pressure, lipids, or glycaemic traits is limited and mixed. Some meta-analyses of RCTs have found sugar intake raised blood pressure and lipids^18^ but others showed no effect on blood pressure^19,20^ or lipids^20,21^ when sugars were substituted isoenergetically for other carbohydrates. Adding fructose-containing sugars to the diet appears to have a harmful effect on fasting insulin but not when substituting sugar for other macronutrients with differences by food sources^22^.

Observationally it is challenging to disentangle the role of a single dietary component, such as sugar, on CVD, while long-term RCTs have not been conducted. In this situation, Mendelian randomization provides an alternative means of examining causal effects by using genetic instruments predicting the exposures to identify effects of single items without confounding. Here, we used two-sample Mendelian randomization^23^ to estimate the effects of blood sucrose^24^, which may reflect sucrose intake because blood sucrose is mainly of dietary origin and metabolized very slowly^25^, on CHD and its risk factors, including T2D, adiposity, blood pressure, lipids, and glycaemic traits using the largest available genome-wide association studies (GWAS). We similarly used blood fructose (one of the two monosaccharides that form sucrose)^26^ as a validation exposure, which we expected to have directionally similar estimates as sucrose. We did not consider blood glucose as an exposure because blood glucose reflects a number of physiological processes as well as dietary intake of sugar and starch. As sugar is known to cause dental caries^1^, we used it as a positive control outcome.

## Results

We obtained 9 and 2 uncorrelated (*r*^2^ < 0.001) single nucleotide polymorphisms (SNP) strongly (*P* < 5 × 10^–6^) predicting blood sucrose and fructose, respectively (Supplementary Table 1). Of the 9 SNPs for blood sucrose, only 2, 3 and 8 were available for lipids in the Global Lipids Genetics Consortium (GLGC), glycaemic traits, and blood pressure, respectively, while all were available for other outcomes. We used rs3821975 as a proxy for rs4693441 (*r*^2^ = 1.0) for lipids in GLGC and glycaemic traits, but the other SNPs had no proxy, giving 3, 4, and 8 SNPs for lipids in GLGC, glycaemic traits, and blood pressure, and 9 SNPs for all other outcomes. Both of the 2 SNPs for blood fructose were available for all outcomes. The 9 and 2 SNPs explained 6.2% and 0.6% of the variance in blood sucrose and fructose, respectively. The mean F-statistic was 15.7 and 23.6, respectively. None of the SNPs was related to key confounders in the UK Biobank or had known pleiotropic effects according to *Ensembl* or *PhenoScanner*.

Genetically higher blood sucrose was positively associated with dental caries, but this association did not remain after accounting for multiple testing (Table 1). Genetically higher blood sucrose was suggestively positively associated with decayed and filled tooth surfaces divided by tooth surfaces (DFSS) but not with decayed, missing and filled tooth surfaces (DMFS). A directionally similar estimate with a much wider confidence interval (CI) was found for blood fructose with dental caries (Supplementary Table 2). Genetically instrumented blood sucrose was not significantly associated with CHD but the direction was positive, as it was for fructose (Table 1 and Supplementary Table 2). Genetically instrumented blood sucrose or fructose were not associated with T2D (Table 1 and Supplementary Table 2), adiposity, blood pressure, lipids, or glycaemic traits (Table 2 and Supplementary Table 3) using any method, with estimates in an inverse direction for adiposity, blood pressure and lipids.

**Table 1:** Associations of genetically predicted blood sucrose (per log_10_ transformed median-normalized SD increase) with dental caries [from the UK Biobank (*n*_case_ = 2110, *n*_control_ = 359,084)], DFSS and DMFS [from the GLIDE consortium, *n* =  ~ 26,792], CHD [from a meta-analysis in the CARDIoGRAMplusC4D consortium (*n*_case_ =  ~ 76,014, *n*_control_ =  ~ 264,785)] and T2D [from a meta-analysis in the DIAGRAM Consortium (*n*_case_ = 74,124, *n*_control_ = 824,006)].

Outcome	Method	SNP	OR/Beta^a^	95% CI	P	P for intercept	Q	P for heterogeneity	P for Global Test
Dental caries	IVW	9	1.04	1.002, 1.08	0.04		6.7	0.57	
	WM	9	1.02	0.97, 1.07	0.55				
	Egger	9	1.02	0.96, 1.09	0.51	0.54			
	PRESSO	9	1.04	1.01, 1.08	0.02				0.59
DFSS (SD change)	IVW	9	0.010	− 0.001, 0.021	0.08		2.8	0.95	
	WM	9	0.008	− 0.006, 0.023	0.26				
	Egger	9	0.006	− 0.013, 0.026	0.53	0.67			
	PRESSO	9	0.010	0.003, 0.016	0.003				0.95
DMFS (SD change)	IVW	9	0.000	− 0.013, 0.014	0.95		11.8	0.16	
	WM	9	0.003	− 0.012, 0.017	0.73				
	Egger	9	− 0.018	− 0.038, 0.002	0.08	0.03			
	PRESSO	9	0.000	− 0.013, 0.014	0.95				0.23
CHD	IVW	9	1.01	0.997, 1.02	0.14		6.6	0.58	
	WM	9	1.01	0.99, 1.02	0.26				
	Egger	9	1.00	0.98, 1.02	0.79	0.17			
	PRESSO	9	1.01	0.998, 1.02	0.14				0.65
T2D	IVW	9	1.00	0.99, 1.01	0.97		3.8	0.88	
	WM	9	1.00	0.99, 1.02	0.44				
	Egger	9	1.00	0.98, 1.01	0.87	0.82			
	PRESSO	9	1.00	0.99, 1.01	0.95				0.88

*CHD* coronary heart disease, *CI* confidence interval, *DFSS* decayed and filled tooth surfaces divided by tooth surfaces, *DMFS* decayed, missing and filled tooth surfaces, *Egger* MR-Egger, *IVW* inverse variance weighting, *OR* odds ratio, *PRESSO* MR-PRESSO, *SD* standard deviation, *SNP* single nucleotide polymorphism, *T2D* type 2 diabetes, *WM* weighted median.

^a^Beta for DFSS and DFMS and OR for other outcomes.

**Table 2:** Associations of genetically predicted blood sucrose (per log_10_ transformed median-normalized SD increase) with adiposity [BMI from a meta-analysis of the GIANT Consortium and the UK Biobank (*n* =  ~ 806,834), WC and BFP from the UK Biobank (*n* =  ~ 361,194)], blood pressure [from a meta-analysis of the UK Biobank and ICBP (*n* =  ~ 757,601)], lipids [from the UK Biobank (*n* =  ~ 361,194) and GLGC (*n* =  ~ 188,578)], and glycaemic traits [from the MAGIC Consortium (*n* =  ~ 46,186)].

Outcome	Method	SNP	Beta	95% CI	P	P for intercept	Q	P for heterogeneity	P for Global Test
BMI (kg/m^2^, SD change)	IVW	9	− 0.003	− 0.007, 0.001	0.15		25.3	0.001	
	WM	9	− 0.004	− 0.007, 0.000	0.04				
	Egger	9	− 0.002	− 0.010, 0.006	0.59	0.80			
	PRESSO	9	− 0.003^a^	− 0.006, − 0.00004	0.05				0.01
WC (cm, SD change)	IVW	9	− 0.001	− 0.005, 0.004	0.76		22.2	0.005	
	WM	9	0.000	− 0.003, 0.004	0.93				
	Egger	9	0.000	− 0.008, 0.009	0.91	0.73			
	PRESSO	9	0.001^a^	− 0.002, 0.003	0.63				0.01
BFP (%, SD change)	IVW	9	− 0.002	− 0.006, 0.002	0.39		23.3	0.003	
	WM	9	− 0.001	− 0.004, 0.002	0.57				
	Egger	9	− 0.001	− 0.008, 0.007	0.85	0.75			
	PRESSO	9	− 0.001^a^	− 0.004, 0.002	0.67				0.01
SBP (mmHg)	IVW	8	0.013	− 0.046, 0.071	0.67		14.3	0.05	
	WM	8	0.017	− 0.047, 0.080	0.61				
	Egger	8	− 0.027	− 0.129, 0.074	0.60	0.34			
	PRESSO	8	0.013	− 0.046, 0.071	0.68				0.03
DBP (mmHg)	IVW	8	− 0.009	− 0.038, 0.021	0.56		11.2	0.13	
	WM	8	− 0.008	− 0.041, 0.026	0.65				
	Egger	8	− 0.008	− 0.063, 0.047	0.77	0.99			
	PRESSO	8	− 0.009	− 0.038, 0.021	0.58				0.14
HDL-C (UKB) (quantile, SD change)	IVW	9	0.000	− 0.003, 0.003	0.96		8.6	0.38	
	WM	9	− 0.001	− 0.004, 0.003	0.79				
	Egger	9	− 0.001	− 0.006, 0.005	0.81	0.80			
	PRESSO	9	0.000	− 0.003, 0.003	0.96				0.42
HDL-C (GLGC) (SD change)	IVW	3	− 0.008	− 0.030, 0.014	0.47		3.2	0.20	
	WM	3	− 0.014	− 0.078, 0.050	0.67				
	Egger	3	− 0.038	− 0.075, − 0.001	0.05	0.07			
LDL-C (UKB) (quantile, SD change)	IVW	9	− 0.001	− 0.004, 0.002	0.40		8.8	0.36	
	WM	9	− 0.001	− 0.005, 0.003	0.67				
	Egger	9	− 0.001	− 0.007, 0.005	0.71	0.93			
	PRESSO	9	− 0.001	− 0.004, 0.002	0.43				0.32
LDL-C (GLGC) (SD change)	IVW	3	0.008	− 0.011, 0.027	0.44		0.1	0.95	
	WM	3	0.008	− 0.056, 0.072	0.81				
	Egger	3	0.004	− 0.037, 0.044	0.86	0.83			
Triglyceride (UKB) (quantile, SD change)	IVW	9	− 0.002	− 0.005, 0.001	0.29		9.2	0.32	
	WM	9	− 0.002	− 0.006, 0.002	0.36				
	Egger	9	− 0.004	− 0.010, 0.001	0.13	0.27			
	PRESSO	9	− 0.002	− 0.005, 0.001	0.32				0.41
Triglyceride (GLGC) (SD change)	IVW	3	0.004	− 0.022, 0.030	0.76		4.9	0.08	
	WM	3	0.005	− 0.093, 0.102	0.93				
	Egger	3	0.026	− 0.036, 0.089	0.40	0.42			
Fasting insulin (pmol/L, log-transformed)	IVW	4	0.003	− 0.009, 0.015	0.64		0.8	0.86	
	WM	4	0.003	− 0.013, 0.020	0.69				
	Egger	4	0.011	− 0.012, 0.035	0.35	0.42			
	PRESSO	4	0.003	− 0.003, 0.009	0.35				0.89
HOMA-B (log-transformed)	IVW	4	0.001	− 0.009, 0.012	0.83		1.6	0.66	
	WM	4	0.003	− 0.012, 0.019	0.68				
	Egger	4	0.012	− 0.009, 0.032	0.26	0.24			
	PRESSO	4	0.001	− 0.006, 0.009	0.78				0.72
HOMA-IR (log-transformed)	IVW	4	0.003	− 0.009, 0.016	0.61		1.4	0.71	
	WM	4	0.003	− 0.014, 0.020	0.69				
	Egger	4	0.013	− 0.011, 0.038	0.29	0.35			
	PRESSO	4	0.003	− 0.005, 0.012	0.47				0.71

*BFP* body fat percentage, *BMI* body mass index, *CI* confidence interval, *DBP* diastolic blood pressure, *Egger* MR-Egger, *GLGC* the Global Lipids Genetics Consortium, *HDL-C* high-density lipoprotein cholesterol, *IVW* inverse variance weighting, *LDL-C* low-density lipoprotein cholesterol, *PRESSO* MR-PRESSO, *SBP* systolic blood pressure, *SD* standard deviation, *SNP* single nucleotide polymorphism, *UKB* UK Biobank, *WC* waist circumference, *WM* weighted median.

^a^Outlier-corrected in MR-PRESSO.

## Discussion

Our Mendelian randomization study suggests a positive association of genetically higher blood sucrose with dental caries, but this association did not withstand allowing for multiple testing. We found no significant association of genetically predicted sucrose with CHD, but a small effect of sucrose on CHD cannot be excluded. Genetically predicted blood sucrose was not associated with risk of T2D, adiposity, blood pressure, lipids, or glycaemic traits. No associations were found for blood fructose.

Our finding of no significant association of blood sucrose with risk of CHD is inconsistent with some meta-analyses of observational studies suggesting a positive association of SSB with CHD^8^ or myocardial infarction^27^ and an observational study suggesting a positive association of sucrose intake with coronary events^28^. However, the observed associations in Western populations may be confounded by socioeconomic position, diet and lifestyle marked by SSB consumption^8,27^. In contrast, a Japanese cohort of 39,786 participants followed up for 18 years suggested no association of soft drink consumption with CHD^29^. Nevertheless, our finding of a non-significant positive association might be owing to inadequate power. Further Mendelian Randomization studies with stronger instruments for sugar intake or biomarkers would be very helpful about the specific role of sugar. More broadly genetic evidence seems to be emerging suggesting that protein rather than fat or sugar is the key dietary element affecting health^30^.

No association was found of genetically predicted blood sucrose or fructose with T2D, which is consistent with a meta-analysis of observational studies of sugar or fructose intake^31^ but not with others suggesting positive associations of SSB with T2D^9,32^. However, the studies of SSB also found positive associations of artificially sweetened beverages with T2D^9,32^, implying that the associations might be due to confounding rather than to sugar itself. The null association with adiposity is concordant with our previous finding of SSB with adiposity in Hong Kong Chinese children^16^, but inconsistent with positive associations of sugar or SSB intake with weight gain in observational studies in Western populations^10,11^. The finding is more consistent with meta-analyses of RCTs showing no effect of substituting sugar for other carbohydrates on weight^10,17,20^. Sucrose or fructose intake, particularly in the form of SSB, may cause adiposity by adding to extra energy intake but inducing less satiety^33^ thus promoting positive energy balance. However, an experimental study in mice found no difference in adiposity by sucrose intake but did find differences by fat intake^34^, suggesting that fat rather than sucrose drives over-consumption. Sensitivity analyses suggested a possible inverse association of blood sucrose with BMI, which might be chance or because sucrose might be at the expense of a lower fat intake. The lack of associations with blood pressure or lipids is consistent with meta-analyses of trials substituting sugar for complex carbohydrates on systolic blood pressure (SBP) and diastolic blood pressure (DBP)^20^ and of trials substituting fructose for glucose on triglyceride^35^, but inconsistent with another showing positive effects of sugar intake on blood pressure and all lipids with or without energy control^18^. However, our MR findings differ from observed positive associations of SSB with hypertension^12^, triglycerides and LDL-C^13^. The lack of associations with glycaemic traits is consistent with a meta-analysis of RCTs showing no effect of sugar intake on fasting insulin when substituting sugar for other macronutrients or subtracting sugar from the diet^22^. Nevertheless, most of the trials had small sample sizes and short duration^18,20,22,35^, making the findings less certain.

Sugar may cause CVD through several mechanisms. First, sugar may promote CVD through its effect on weight gain. Observational studies have suggested a weakened association of SSB with CHD after adjusting for body mass index (BMI) and energy intake^8^. However, while sugar at high doses in hypercaloric trials did increase weight^17^, sugar at usual intake especially as part of an isocaloric diet showed no effect on weight gain^10,17^. Second, sugar may cause CVD through metabolic effects of fructose. Unlike glucose, fructose can bypass phosphofructokinase and be metabolised to substrates for lipid synthesis in unregulated glycolysis^36^, thus leading to increased de novo lipogenesis, dyslipidaemia, and insulin resistance after excess intake^36^. Chronic fructose intake may activate gene expression of lipogenic enzymes to further enhance lipogenesis^36^. Unregulated fructose metabolism can also raise uric acid concentration, which has been linked to endothelial dysfunction, hypertension, and increased risk of CVD^33^. It is also possible that sugar intake might affect glucagon or insulin which may cause CHD^37,38^. However, we did not find any evidence of these pathways, which need validation using stronger instruments predicting sugar.

Our study has some limitations. First, we used genetic variants predicting sucrose and fructose from metabolomics studies using mass spectrometry^24,26^. Blood sucrose and fructose may not be perfect biomarkers of sugar intake. However, blood sucrose is mainly of dietary origin and metabolized very slowly^25^, so blood sucrose reflects sucrose intake. High fructose corn syrup (42% or 55% fructose, glucose and water) is another commonly used sweetener other than sucrose, which may be captured by blood fructose. Many other factors, including intestinal permeability and liver metabolism^39^, may also influence blood levels of sucrose and fructose, but are unlikely to confound the genetic associations. Second, we used the genetic variants associated with blood sucrose and fructose at less than genome-wide significance (*P* < 5 × 10^–6^) and the variance of sucrose and fructose explained by the genetic variants were small (6.2% and 0.6%). Weak instrument bias could attenuate the causal estimates towards the null^23^. However, the SNPs had a mean F-statistic > 10. Weak instrument bias is expected to be small if the p-value is below 1 × 10^–5^^40^. Third, confounding by population stratification is possible. However, the genetic associations concern people of European ancestry generated with genomic control. The GWAS of blood sucrose suggested no inflation or deflation due to population stratification^24^. The genetic variants were not related to key confounders in the UK Biobank. Fourth, genetic predictors of sucrose and fructose may have pleiotropic effects. However, we found no unknown pleiotropy from MR-Egger and MR-PRESSO and no known pleiotropy in *Ensembl* or *PhennoScanner*. All methods gave similar results. Fifth, our study was mainly limited to people of European ancestry and might not apply to other populations. However, causes would be expected to be consistent^41^, although might not always be relevant^42^. Sixth, the instruments predicting blood sucrose were obtained from primarily women, which may not be applicable to men. However, there is no evidence that genetic predisposition to blood sucrose or fructose differs by sex or has different effects by sex. The estimates for blood sucrose or fructose with dental caries, BMI and blood pressure in women were similar to those in both sexes. Seventh, we could not check whether the associations varied by age, or for non-linear effects. Eighth, canalization, i.e., compensatory mechanisms driving the association of genetic variants with sucrose or fructose, may result in different associations in Mendelian randomization than would be observed from interventions. However, whether that exists is unknown. Finally, although the estimates were close to the null, we cannot rule out a small effect for CHD, which should be investigated further. We only have 80% power to detect an OR of about 1.05 for sucrose on CHD. However, the estimates for adiposity, blood pressure and lipids were in the opposite direction to those expected.

## Conclusions

Our study found some evidence of a detrimental effect of blood sucrose on dental caries. However, our study suggests no effect of blood sucrose on T2D, adiposity, blood pressure, lipids, or glycaemic traits, whilst it remains possible that there could be a small effect of sucrose on CHD risk.

## Methods

### Genetic instruments for blood sucrose and fructose

Genetic variants, i.e., SNP, strongly (*P* < 5 × 10^–6^) predicting serum sucrose (log_10_ transformed median-normalized values) were extracted from an existing GWAS of serum metabolites adjusted for sex and age, obtained from non-targeted metabolomics using four independent ultra-high-performance liquid chromatography-tandem mass spectrometry^24^, in 1439 individuals (median age 58 years, 96.6% women) of European ancestry^24^. These associations were not adjusted for principal components because there was no evidence of inflation or deflation due to population stratification^24^. SNPs strongly (*P* < 5 × 10^–6^) predicting blood fructose (log_10_ transformed normalized) were extracted from an existing GWAS of blood metabolites adjusted for age, sex, batch and genomic control^26^, obtained using liquid-phase chromatography, gas chromatography separation and tandem mass spectrometry^26^ in 7824 individuals (mean age 55 years, 83.5% women) of European ancestry^26^. SNPs were discarded if they were non-biallelic or had a minor allele frequency less than 0.01.

### Genetic associations with dental caries, CHD, and T2D

Genetic associations with dental caries (*n*_case_ = 2110, *n*_control_ = 359,084) were extracted from genetic summary statistics from the UK Biobank in ~ 361,194 white British individuals (mean age 57.5 years)^43^ provided by Neale Lab (http://www.nealelab.is/uk-biobank/). Dental caries was defined as hospital inpatient admission due to International Classification of Diseases (ICD) 10 K02. These genetic associations were obtained from linear regresstion adjusted for age, age^2^, sex, interactions of sex with age and age^2^, and 20 principal components. The linear regression estimates were transformed to log odds ratios (OR) using an approximation^44^. Genetic associations with DFSS (inverse normal rank transformed) and DMFS (standard deviation of residuals) were taken from summary statistics from the Gene-Lifestyle Interactions in Dental Endpoints (GLIDE) consortium in up to 26,792 individuals of European ancestry adjusted for age, sex, and study-specific covariates^45^. Genetic associations with CHD were taken from summary statistics of the Coronary Artery DIsease Genome wide Replication and Meta-analysis plus Coronary Artery Disease (CARDIoGRAMplusC4D) consortium from a meta-analysis (*n*_case_ =  ~ 76,014, *n*_control_ =  ~ 264,785) of CARDIoGRAMplusC4D 1000 Genomes, the Myocardial Infarction Genetics and CARDIoGRAM Exome, the UK Biobank SOFT CAD GWAS, and two small case–control studies in individuals of mainly European ancestry (77% in CARDIoGRAMplusC4D 1000 Genomes and 94% in the UK Biobank SOFT CAD GWAS)^46^ with genetic control. CHD cases included fatal or nonfatal myocardial infarction, percutaneous transluminal coronary angioplasty, coronary artery bypass grafting, chronic ischemic heart disease and angina determined from clinical diagnosis, medical records and self-report of medication usage. Controls were those who were not cases. Genetic associations with T2D were obtained from summary statistics from the DIAbetes Genetics Replication And Meta-analysis (DIAGRAM) consortium from a meta-analysis of case (*n* = 74,124)-control (*n* = 824,006) studies^47^ in individuals of European ancestry adjusted for study-specific covariates and population structure but not BMI.

### Genetic associations with adiposity, blood pressure, lipids, and glycaemic traits

Genetic associatons with inverse-normally transformed BMI were obtained from summary statitsics from a meta-analysis of the Genetic Investigation of ANthropometric Traits (GIANT) Consortium and the UK Biobank in up to 806,834 individuals of European ancestry adjusted for age, age^2^, assessment centre, and sex^48^. Genetic associations with inverse-rank normalized waist circumference (WC), body fat percentage (BFP), high-density lipoprotein cholesterol (HDL-C), LDL-C, and triglyceride were obtained from UK Biobank summary statitsics^43^ provided by Neale Lab (http://www.nealelab.is/uk-biobank/) in ~ 361,194 white British individuals adjusted for age, age^2^, and 20 principal components, sex and interactions of sex with age and age^2^. Genetic associations with inverse-normally transformed HDL-C, LDL-C, and triglyceride were also taken from GLGC in ~ 188,578 individuals of European ancestry adjusted for age, age^2^, and sex^49^. Genetic associations with SBP and DBP were obtained from a meta-analysis of the UK Biobank and the International Consortium of Blood Pressure-Genome Wide Association Studies (ICBP) in ~ 757,601 individuals of European ancestry adjusted for sex, age, age^2^, BMI, and genotyping chips^50^. Genetic associations with glycaemic traits, including indices of β-cell function (HOMA-B) and insulin resistance (HOMA-IR), and fasting insulin (all naturally log transformed), were taken from a meta-analysis of GWAS including the UK Biobank in up to 46,186 individuals of European ancestry adjusted for age, sex and study specific covariates with genomic control^51^ provided by the Meta-Analyses of Glucose and Insulin-related traits Consortium (MAGIC).

### Statistical analysis

We obtained uncorrelated SNPs (*r*^2^ ≤ 0.001) using the “ld_clump” function of “ieugwasr” R package. For SNPs not available for an outcome, we used proxies (*r*^2^ ≥ 0.8) identified from *LDlink* (https://analysistools.nci.nih.gov/LDlink/). We aligned genetic variants for exposure and outcome on effect allele as none were palindromic. We estimated the F-statistic for each SNP using an approximation^52^, where a F-statistic of 10 or above indicated a lower risk of weak instrument bias. We checked whether any of the SNPs were associated with key confounders (i.e., Townsend deprivation index, education, smoking, alcohol drinking, and moderate physical activity) in the UK Biobank at Bonferroni corrected significance (0.05 divided by the number of SNPs times the number of confounders) (http://www.nealelab.is/uk-biobank). We checked for known pleiotropic effects using two curated genotype-to-phenotype cross-references, *Ensembl 99* (http://www.ensembl.org/index.html, accessed April 15, 2020), and *PhenoScanner* (http://www.phenoscanner.medschl.cam.ac.uk, accessed April 15, 2020).

We obtained Mendelian randomization estimates by meta-analysing SNP-specific Wald estimates (ratio of SNP on outcome to SNP on exposure)^23^ using inverse variance weighting (IVW) with multiplicative random effects which allows for heterogeneity but assumes balanced pleiotropy. We also used a weighted median which is valid as long as < 50% of the weight are contributed by invalid SNPs^53^, and MR-Egger which is valid as long as the instrument strength independent of direct effect assumption (i.e., the size of the pleiotropic effects is independent of their strength as instruments) holds^52^. MR-Egger provides a check on the validity of the IVW estimate, with a significant p-value (< 0.05) for the intercept indicating that the IVW estimate may be invalid^52^. We also used the Mendelian randomization pleiotropy residual sum and outlier (MR-PRESSO) test which can correct for potential horizontal pleiotropy (i.e., the variant has an effect on disease outside of its effect on the exposure)^54^.

We estimated the expected effect size of the association of blood sucrose with CHD for 80% power. At an R^2^ (the proportion of variance in blood sucrose explained by the genetic variants) of 6.2%, we have 80% power to detect an OR of ∼1.05 for sucrose on CHD. Here, we are testing a hypothesis, the effect on CHD with related secondary outcomes (CHD risk factors) and a control outcome (dental caries), for comprehensiveness we considered a p-value of 0.05 and a Bonferroni-corrected p-value of 0.05/7 = 0.007 (given 7 outcomes including CHD, 5 CHD risk factors and dental caries) as significant. All statistical analyses were performed using R version 3.6.3 (The R Foundation for Statistical Computing, Vienna, Austria)^55^ and the “MendelianRandomization” and “MRPRESSO” R packages. This study requires no ethical approval because only publicly available summary data was used.

## Supplementary Information

Supplementary Information.

## Data Availability

The data are available in the consortia.

## Abbreviations

BFP: Body fat percentage
BMI: Body mass index
CARDIoGRAMplusC4D: The Coronary Artery DIsease Genome wide Replication and Meta-analysis plus Coronary Artery Disease consortium
CHD: Coronary heart disease
CI: Confidence interval
DBP: Diastolic blood pressure
DFSS: Decayed and filled tooth surfaces divided by tooth surfaces
DIAGRAM: The DIAbetes Genetics Replication And Meta-analysis consortium
DMFS: Decayed, missing and filled tooth surfaces
GLIDE: The Gene-Lifestyle Interactions in Dental Endpoints consortium
GLGC: The Global Lipids Genetics Consortium
GWAS: Genome-wide association study
HDL-C: High-density lipoprotein cholesterol
ICBP: International Consortium of Blood Pressure-Genome Wide Association Studies
ICD: International Classification of Diseases
IVW: Inverse variance weighting
LDL-C: Low-density lipoprotein cholesterol
MAGIC: The Meta-Analyses of Glucose and Insulin-related traits Consortium
OR: Odds ratio
RCT: Randomized controlled trial
SBP: Systolic blood pressure
SD: Standard deviation
SNP: Single nucleotide polymorphism
SSB: Sugar-sweetened beverage
T2D: Type 2 diabetes
WC: Waist circumference
WHO: World Health Organization
