# Supplementary material for: Association of genetically predicted blood sucrose with coronary heart disease and its risk factors in Mendelian randomization
Source: Sci Rep. 2020 Dec 9;10:21588. doi: 10.1038/s41598-020-78685-5 (PMC7725802; doi:10.1038/s41598-020-78685-5)
Supplement: Supplementary file 1 — Supplementary Information. [file 41598_2020_78685_MOESM1_ESM.pdf]

SUPPLEMENTAL MATERIAL

for

Association of genetically predicted blood sucrose with coronary heart disease and its risk factors in Mendelian randomization

Ting Zhang[1]; Shiu Lun Au Yeung[1], C. Mary Schooling[1, 2]

1. School of Public Health, Li Ka Shing Faculty of Medicine, The University of Hong Kong, Hong Kong SAR, China

2. CUNY School of Public Health and Health Policy, New York, USA

Supplementary Material

Supplementary Table 1 SNPs used in the Mendelian randomization analysis of the associations of blood sucrose and fructose with each outcome.

Exposure	SNP	Nearest Gene	Position	EA/OA	EAF	F	Exposure			Dental caries			DFSS			DMFS		
							Beta	SE	P	Beta†	SE	P	Beta	SE	P	Beta	SE	P
Blood	rs116805764	UBE2E2	3:23077604	T/C	0.03	15.8	1.492784	0.376078	4.04e-06	-0.00965	0.100568	0.929972	-0.0085	0.035	0.8077	-0.0761	0.0346	0.02766
sucrose	rs143349171	ZNF804A	2:183986330	A/C	0.04	14.4	1.05145	0.276872	1.67e-06	0.074804	0.087157	0.3978	0.0086	0.0338	0.7981	0.0268	0.0336	0.4249
	rs1593306	PLXNA4	7:131929204	A/C	0.92	4.4	0.742769	0.354395	2.77e-06	0.075242	0.062528	0.23104	-0.0026	0.0157	0.8661	-0.0017	0.0157	0.9150
	rs4693441	ENOPH1	4:82465026	C/T	0.45	18.7	0.218304	0.050446	4.31e-06	0.027476	0.029942	0.364865	0.0088	0.009	0.3318	0.0195	0.009	0.03084
	rs60172187	RAB31	18:9734087	T/G	0.05	17.3	1.681877	0.404894	1.85e-06	0.003419	0.063678	0.961084	0.0224	0.018	0.2119	0.0073	0.018	0.6851
	rs6944685	SEMA3D	7:85727431	T/C	0.31	14.1	0.585664	0.156242	3.36e-06	0.005978	0.031834	0.861299	0.0048	0.0101	0.6361	0.0057	0.0101	0.5738
	rs7151852	TMEM229B	14:67478305	A/C	0.03	17.7	1.265056	0.300583	2.04e-06	0.191607	0.079004	0.01521	0.0300	0.018	0.09629	0.0112	0.0176	0.5234
	rs72860869	SPSB1	1:9344308	G/T	0.07	20.8	1.430037	0.313844	5.09e-07	0.076394	0.053471	0.153581	0.0082	0.0161	0.6100	-0.0122	0.0162	0.4526
	rs80233112	AHR	7:17180858	C/A	0.03	18.4	1.420512	0.331573	1.20e-06	-0.00462	0.087443	0.961688	-0.0068	0.0437	0.8765	-0.0184	0.0437	0.6733
Blood	rs2456584	C19orf48	19:56004957	A/G	0.54	23.4	-0.0179	0.0037	1.64e-06	-0.00662	0.029893	0.83603	0.0067	0.0088	0.4409	0.0012	0.0087	0.8937
fructose	rs661454	TFAP2D	6:50728637	A/G	0.08	23.8	0.0317	0.0065	1.31e-06	0.07417	0.05007	0.138766	-0.0182	0.0142	0.2019	-0.0115	0.0144	0.4231

DFSS, decayed and filled tooth surfaces divided by tooth surfaces; DMFS, decayed, missing and filled tooth surfaces; EAF, effect allele frequency; EA/OA: effect allele/other allele; SE, standard error; SNP, single nucleotide polymorphism.
† transformed log odds ratio using an approximation.

Continued

Exposure	SNP	EA/OA	CHD			T2D			BMI			WC			BFP		
			**Beta**	**SE**	**P**	**Beta**	**SE**	**P**	**Beta**	**SE**	**P**	**Beta**	**SE**	**P**	**Beta**	**SE**	**P**
Blood	rs116805764	T/C	0.01694	0.02957	0.566811	-0.019	0.021	0.38	0.0143	0.0064	0.02449	0.0113875	0.00671076	0.0897157	0.00923202	0.00578269	0.11038
sucrose	rs143349171	A/C	0.05469	0.03059	0.073784	0.0032	0.018	0.86	-0.0156	0.0059	0.00851	-0.0100621	0.00631381	0.11101	-0.0113493	0.0054474	0.0372121
	rs1593306	A/C	-0.00481	0.0165	0.770552	-0.0083	0.013	0.52	-0.0113	0.0033	0.00054	-0.0160658	0.00403775	6.92e-05	-0.0128564	0.00348097	0.00022135
	rs4693441	C/T	0.01168	0.00823	0.155651	0.0049	0.0064	0.45	0.0004	0.0019	0.82	0.00083417	0.00206728	0.686571	0.00096055	0.00178244	0.589958
	rs60172187	T/G	0.01356	0.01695	0.423903	0.0077	0.014	0.58	-0.0068	0.0041	0.09228	0.00038437	0.00441531	0.930628	-0.0017244	0.00380661	0.650554
	rs6944685	T/C	0.01136	0.00938	0.225827	-0.0026	0.0068	0.7	-0.0018	0.0019	0.344	0.00014979	0.00220471	0.945831	-0.001477	0.00190106	0.437208
	rs7151852	A/C	0.01478	0.02154	0.492583	-0.019	0.02	0.34	-0.0050	0.0052	0.3382	-0.0020563	0.00648019	0.750996	-0.0083459	0.00558804	0.135301
	rs72860869	G/T	-0.00357	0.01743	0.837617	0.0081	0.013	0.52	-0.0048	0.0035	0.1706	-0.0002411	0.00392876	0.951068	-0.0009774	0.00338821	0.772995
	rs80233112	C/A	-0.02764	0.03641	0.447847	0.0084	0.019	0.66	0.0071	0.0056	0.206	0.00564191	0.00596852	0.344518	0.00626055	0.00514327	0.223517
Blood	rs2456584	A/G	-0.00564	0.00825	0.494588	0.003	0.0062	0.63	0.0031	0.0017	0.06837	0.0014549	0.00206733	0.481583	0.00161502	0.0017825	0.364914
fructose	rs661454	A/G	0.00416	0.01393	0.765203	0.005	0.011	0.66	-0.0011	0.0028	0.7014	-0.000941	0.00366787	0.797534	-0.0022476	0.00316233	0.477239

EAF, effect allele frequency; EA/OA: effect allele/other allele; SE, standard error; SNP, single nucleotide polymorphism; CHD, coronary heart disease; T2D, type 2 diabetes; BFP, body fat percentage; BMI, body mass index; WC, waist circumference.

Continued

Exposure	SNP	EA/OA	SBP			DBP			HDL-C (UKB)			LDL-C (UKB)			Triglyceride (UKB)		
			Beta	SE	P	Beta	SE	P	Beta	SE	P	Beta	SE	P	Beta	SE	P
Blood	rs116805764	T/C	-0.0714	0.0987	0.4697	0.0646	0.0565	0.2534	-0.014645	0.0073999	0.04781	-0.0053102	0.0076431	0.4872	0.0085775	0.0075302	0.25467
sucrose	rs143349171	A/C	-0.1697	0.1003	0.0906901	-0.1399	0.0569	0.0139399	0.00090252	0.0069704	0.89698	0.0056115	0.0072057	0.43612	-0.0040077	0.0070997	0.57242
	rs1593306	A/C	-0.041	0.0605	0.4984	-0.0432	0.0346	0.2121	0.0077233	0.0044531	0.082852	-0.001551	0.0046057	0.7363	-0.0084673	0.0045383	0.062081
	rs4693441	C/T	0.0172	0.0304	0.571	-0.001	0.0174	0.9555	-7.529e-05	0.0022782	0.97364	-0.0033348	0.0023568	0.15708	0.0026402	0.002322	0.25553
	rs60172187	T/G	-0.0734	0.0639	0.2504	-0.0449	0.0369	0.2238	0.0023503	0.0048711	0.62945	-0.0011344	0.0050368	0.82181	-0.002335	0.0049621	0.63795
	rs6944685	T/C	0.0798	0.0337	0.0178999	0.0066	0.0192	0.731301	-0.0005264	0.0024323	0.82866	0.0035397	0.0025146	0.15923	0.0014305	0.0024776	0.56368
	rs7151852	A/C	-	-	-	-	-	-	-0.0043692	0.0071463	0.54094	-0.0003139	0.0073945	0.96614	-0.010834	0.0072858	0.13703
	rs72860869	G/T	0.1086	0.06	0.07057	0.0333	0.0342	0.3309	-0.0019452	0.0043332	0.6535	-0.0092536	0.0044767	0.038729	-0.0030821	0.0044104	0.48466
	rs80233112	C/A	0.0586	0.0993	0.555199	-0.0322	0.0562	0.5662	0.0059566	0.006566	0.36431	0.0014222	0.0067983	0.83429	-0.0063541	0.0066989	0.34286
Blood	rs2456584	A/G	-0.0062	0.0301	0.8358	-0.0229	0.0174	0.1891	-0.0008317	0.0022791	0.71516	-0.0004484	0.0023577	0.84916	-0.0002139	0.0023229	0.92665
Fructose	rs661454	A/G	-0.1291	0.0534	0.0156401	-0.047	0.0306	0.1249	-0.0050431	0.0040468	0.2127	-0.0039619	0.0041844	0.34373	0.0025608	0.0041226	0.53449

DBP, diastolic blood pressure; EAF, effect allele frequency; EA/OA, effect allele/other allele; HDL-C, high-density lipoprotein cholesterol; LDL-C, low-density lipoprotein cholesterol; SBP, systolic blood pressure; SE, standard error; SNP, single nucleotide polymorphism; UKB, UK Biobank.

Continued

Exposure	SNP	EA/OA	HDL-C (GLGC)			LDL-C (GLGC)			Triglyceride (GLGC)			Fasting Insulin			HOMA-B			HOMA-IR		
			**Beta**	**SE**	**P**	**Beta**	**SE**	**P**	**Beta**	**SE**	**P**	**Beta**	**SE**	**P**	**Beta**	**SE**	**P**	**Beta**	**SE**	**P**
Blood	rs116805764	T/C	-	-	-	-	-	-	-	-	-	-	-	-	-	-	-	-	-	-
sucrose	rs143349171	A/C	-	-	-	-	-	-	-	-	-	-	-	-	-	-	-	-	-	-
	rs1593306	A/C	-0.0135	0.0127	0.4035	0.002	0.0136	0.8639	-0.0074	0.012	0.5552	0.0048	0.012	0.6762	0.0046	0.0097	0.637	0.0079	0.012	0.5152
	rs4693441	C/T	0.0062*	0.0049	0.07443	0.0025*	0.0053	0.6913	-0.0063*	0.0048	0.1842	-0.0017*	0.0038	0.6551	-0.0036*	0.0033	0.2759	-0.0018*	0.004	0.6491
	rs60172187	T/G	-	-	-	-	-	-	-	-	-	-	-	-	-	-	-	-	-	-
	rs6944685	T/C	-0.0078	0.0069	0.3791	0.0052	0.0076	0.5294	0.0117	0.0067	0.1276	0.0009	0.0051	0.8667	0.002	0.0043	0.6394	-0.0004	0.0053	0.939
	rs7151852	A/C	-	-	-	-	-	-	-	-	-	0.013	0.017	0.4343	0.0028	0.016	0.8629	0.018	0.018	0.3329
	rs72860869	G/T	-	-	-	-	-	-	-	-	-	-	-	-	-	-	-	-	-	-
	rs80233112	C/A	-	-	-	-	-	-	-	-	-	-	-	-	-	-	-	-	-	-
Blood	rs2456584	A/G	0.0017	0.0049	0.6575	-0.0071	0.0054	0.2885	-0.0032	0.0048	0.4258	-0.0045	0.0039	0.2491	-8.00E-04	0.0033	0.8165	-0.0061	0.004	0.1343
Fructose	rs661454	A/G	-0.0036	0.0062	0.5032	0	0.0066	0.9632	0.0047	0.0061	0.2263	-0.0093	0.007	0.1841	-0.0096	0.0063	0.1265	-0.012	0.0074	0.1081

EAF, effect allele frequency; EA/OA: effect allele/other allele; GLGC, the Global Lipids Genetics Consortium; SE, standard error; SNP, single nucleotide polymorphism.
* rs4693441 replaced by a proxy rs3821975 (r^2 =1.0).

Supplementary Table 2 Associations of genetically predicted blood fructose (per $\log_{10}$ transformed SD increase, based on independent SNPs with $P<5\times10^{-6}$) with dental caries (from the UK Biobank (n_{case}=2,110, $n_{control}$=359,084)), DFSS and DMFS (from the GLIDE consortium, n=~26,792), CHD (from a meta-analysis in the CARDIoGRAMplusC4D consortium (n_{case}=~76,014, $n_{control}$=~264,785)) and T2D (from a meta-analysis in the DIAGRAM Consortium (n_{case}=74,124, $n_{control}$=824,006)).

Outcome	Method	SNP	OR/Beta*	95% CI	P	Q	P for heterogeneity
Dental caries	IVW	2	4.09	0.43, 38.8	0.22	0.7	0.39
DFSS (SD change)	IVW	2	-0.48	-1.13, 0.17	0.14	0.09	0.76
DMFS (SD change)	IVW	2	-0.23	-0.88, 0.43	0.50	0.20	0.66
CHD	IVW	2	1.24	0.67, 2.32	0.49	0.1	0.77
T2D	IVW	2	0.99	0.62, 1.61	0.98	0.4	0.51

CI, confidence interval; Egger, MR-Egger; IVW, inverse variance weighting; OR, odds ratio; SD, standard deviation; WM, weighted median.
* Beta for DFSS and DFMS and OR for other outcomes.

Supplementary Table 3 Associations of genetically predicted blood fructose (per log_{10} transformed SD increase, based on independent SNPs with $P<5\times10^{-6}$) with adiposity (BMI from a meta-analysis of the GIANT Consortium and the UK Biobank (*n*=~806,834), WC and BFP from the UK Biobank (*n*=~361,194)), blood pressure (from a meta-analysis of the UK Biobank and ICBP (*n*=~757,601)), lipids (from the UK Biobank (*n*=~361,194) and GLGC (*n*=~188,578)), and glycaemic traits (from the MAGIC Consortium (*n*=~46,186)).

Outcome	Method	SNP	Beta	95% CI	P	Q	P for heterogeneity
BMI (kg/m^2, SD change)	IVW	2	-0.099	-0.234, 0.036	0.15	1.1	0.29
WC (cm, SD change)	IVW	2	-0.056	-0.216, 0.105	0.50	0.1	0.75
BFP (%, SD change)	IVW	2	-0.081	-0.219, 0.058	0.25	0.02	0.89
SBP (mmHg)	IVW	2	-1.859	-6.190, 2.471	0.40	3.4	0.06
DBP (mmHg)	IVW	2	-0.111	-2.818, 2.595	0.94	4.1	0.04
HDL-C (UKB) (quantile, SD change)	IVW	2	-0.056	-0.257, 0.145	0.59	1.3	0.25
HDL-C (GLGC) (SD change)	IVW	2	-0.107	-0.419, 0.205	0.50	0.0	0.96
LDL-C (UKB) (quantile, SD change)	IVW	2	-0.050	-0.233, 0.133	0.59	0.6	0.42
LDL-C (GLGC) (SD change)	IVW	2	0.128	-0.235, 0.491	0.49	1.2	0.28
TG (UKB) (quantile, SD change)	IVW	2	0.046	-0.134, 0.226	0.61	0.1	0.71
TG (GLGC) (SD change)	IVW	2	0.159	-0.148, 0.465	0.31	0.0	0.93
Fasting insulin (pmol/L, log-transformed)	IVW	2	-0.017	-0.551, 0.516	0.95	3.1	0.08
HOMA-B (log-transformed)	IVW	2	-0.116	-0.456, 0.224	0.50	1.6	0.20
HOMA-IR (log-transformed)	IVW	2	-0.003	-0.707, 0.701	0.99	5.0	0.03

CI, confidence interval; GLGC, the Global Lipids Genetics Consortium; IVW, inverse variance weighting; SD, standard deviation; UKB, UK Biobank.
